# Differences in parental stress in mothers and fathers of preterm infants: A prospective study in Poland

**DOI:** 10.18332/ejm/200552

**Published:** 2025-03-07

**Authors:** Barbara Zych, Witold Błaż, Katarzyna Kanadys, Ewa Dmoch-Gajzlerska, Małgorzata Nagórska

**Affiliations:** 1 Institute of Health Sciences, Medical College of Rzeszow University, Rzeszow, Poland; 2 Institute of Medical Sciences, Medical College of Rzeszow University, Rzeszow, Poland; 3 Department of Obstetrics and Gynecology Nursing, Medical University of Lublin, Lublin, Poland; 4 Faculty of Health Sciences, Mazovian Academy of Applied Sciences, Siedlce, Poland

**Keywords:** parents, stress, stress management strategies, Kangaroo Mother Care

## Abstract

**INTRODUCTION:**

The birth of a premature baby and hospitalization are stressful events for parents. The study aimed to assess the differences in parental stress experienced by parents of preterm infants provided with Kangaroo Mother Care (KMC) during their stay in the hospital.

**METHODS:**

The cross-sectional study was conducted among 337 randomly chosen parents of hospitalized babies born in Poland in 2016. As research tools, the Personal Information Form (PIF), Parental Stressor Scale: Neonatal Intensive Care Unit (PSS:NICU) and Coping Inventory for Stressful Situations (CISS) were used.

**RESULTS:**

The significant association in every subscale of PSS:NICU (p<0.001 vs p=0.003 vs p<0.001) with the overall stress score (p<0.001) were confirmed. The start time of KMC implementation also turned out to be significantly lower in the group of mothers starting KMC at 1 week of the child’s life for the overall stress score and subscales and I and III (p=0.024 vs p=0.024; total: p=0.005), while KMC did not affect the PSS:NICU results in the group of mothers and fathers. Parents' dominant stress management strategy was Task-Oriented Coping (p=0.123), but confronting the reality with Avoidance-Oriented Coping was the basis of their adaptation (p=0.591).

**CONCLUSIONS:**

The parental stress level was higher in mothers than in fathers. Mothers' dominant strategy of coping with stress was Seeking Social Contact, and in fathers Emotion-Oriented Coping.

## INTRODUCTION

The birth of a premature baby and the associated separation is a source of great stress and guilt for parents, especially for the mother, due to the treatment of their children in the NICU (Neonatal Intensive Care Unit)^1-3^. Endler and Parker^4^ pointed out that stress cannot be avoided and distinguished between the three stress management strategies: Task-Oriented Coping (TOC), Emotion-Oriented Coping (EOC) and Avoidance-Oriented Coping (AOC). They further categorized Avoidance-Oriented Coping into two subscales: engaging in substitute activities (SA) and seeking social contacts (SC). The choice of coping strategy depends on the situation and individual characteristics^5^. We considered a widely recognized method of caring for a premature baby – Kangaroo Mother Care (KMC), which provides skin-to-skin (SSC) contact between a child and a parent in a home environment. A key element of KMC is skin-to-skin contact between the baby and the mother (the optimal KMC provider) because SSC is the basis for early, effective and exclusive breastfeeding^6^. Fathers can also perform kangaroo care among premature babies during a hospital stay or after the child is discharged home^2^. Studies have revealed that in the group of mothers of kangaroo care practicing, there was less anxiety, improved self-esteem and acceptance of the child’s stay in the NICU, as well as greater maternal involvement in child care^7^. Also, it was observed that in fathers who used the kangaroo method, anxiety was gradually reduced, and they found themselves in the role of father more easily^8^; they were also willing to take care of babies and more sensitive to their needs^9^.

Therefore, this study aimed to examine the differences in the experience of stress and the ways of dealing with it by parents of premature babies who, during their stay in the NICU, are provided with KMC.

The questions we are trying to answer are: 1) What are the differences in stress between mothers and fathers of premature babies; 2) What are the differences in coping strategies for mothers and fathers of premature babies; and 3) Are there differences in parental stress and coping strategies of mothers and fathers when providing KMC, i.e. the time of commencement of the first contact of the SSC with the child (up to 1 week of the child’s life vs more than 1 week of the child’s life) and the duration of the SSC session (KMC daily vs KMC with breaks). Achieving our goal will allow us to acquire knowledge about the stress level in parents who have decided to take early care of their child through skin-to-skin contact during KMC.

## METHODS

### Study design and setting

**Figure 1:**
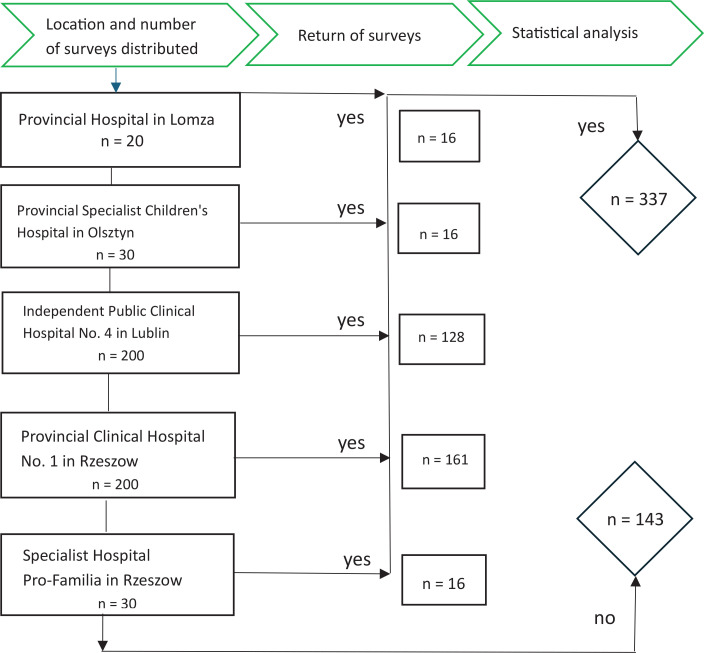
Flow diagram

The cross-sectional study was conducted among 337 parents of preterm babies born in 2016 and hospitalized in the NICU. The study included randomly selected parents, mainly from eastern Poland (Lubelskie, Podkarpackie, and Podlaskie voivodeships, the highest-level administrative divisions of Poland), who gave birth between January and December 2016 and were hospitalized in the NICU due to prematurity (Figure 1). The study size was calculated with a maximum error of 5.0% and a confidence level of 95.0%, which for the size of the studied population was a minimum of 138 people. In our case, the size of the general sample included 382257 live births, of which 44106 were children <2500 g (about 11.5% of the population)^10^. The study’s methodology has been published in detail in our earlier publication^11^.

### Participants

A randomly selected group of participants who met the study inclusion criteria were selected for the study. Inclusion criteria were as follows: the parents of the newborn in gestational age 27–36 weeks and stable clinical condition. The criterion for exclusion from the study was the lack of parental consent to participate in the study and unstable general condition and clinical situations of newborns (respiratory problems including apnea, respiratory effort, SpO2 <90%, flaccid or inactive child).

The essential value of the arrangements made in this area was to ensure the child’s and the parents’ safety. The criteria for including a parent in the study were: parents of preterm born infants hospitalized in NICU and informed consent to participate in the study. All of the parents studied were biological parents and employed kangaroo care to their children individually or both parents in turn. Single-parent families, divorced or separated parents were excluded from the study because the experience might have caused stress resulting in psychological discomfort. After the first contact with kangaroo mother care and skin-to-skin contact between parent and child, the surveyed parents received the Parental Stressor Scale: Neonatal Intensive Care Unit (PSS:NICU) and Coping Inventory for Stressful Situations (CISS) to complete.

### Data sources and measurement

The questionnaire included three parts: the self-administrated part Personal Information Form (PIF) and two standardized questionnaires, Neonatal Intensive Care Unit (PSS:NICU) and the Coping Inventory for Stressful Situations (CISS).

*Personal Information Form (PIF)*

An information questionnaire designed solely for this study was used to collect sociodemographic and medical data of parents and their children. The respondents were asked about: sex, age, the level of education, and clinical data connected with the pregnancy (diseases during pregnancy, hospitalizations and the mode of delivery). The questionnaire regarding the child contained questions about: sex, the birth weight of the child, and general condition measured on the Apgar scale score. The PIF data were collected through interviews with the parents and the Apgar scale score from medical records.

*Parental Stressor Scale: Neonatal Intensive Care Unit (PSS:NICU)*

PSS:NICU is a scale used to assess the level of parental stress. PSS:NICU includes three scales covering sources of stress generated by the hospital environment: sights and sounds related to the therapeutic and diagnostic process (subscale 1), infant appearance and behavior (subscale 2) and parental role alternation during hospitalization and treatment of the baby, the equipment used and the relationship between parents and the staff (subscale 3). The survey includes a total of 34 questions. The degree of stress in subjects was determined on a 5-point Lickert scale (1 – not at all stressful the experience did not cause you to feel upset, tense, or anxious; 2 – a little stressful, 3 – moderately stressful, 4 – very stressful, 5 – extremely stressful). In case the situation described was neutral or not experienced during the child’s hospitalization in NICU, the answer was scored as 0 points. After obtaining the consent of the PSS:NICU questionnaire’s authors (Miles, Funk and Carlson 1993), we evaluated the questionnaire’s validation concerning its practical application in Poland, as described in our earlier article^11^. The internal compliance index Cronbach α for the PSS:NICU questionnaire applied for a group of KMC parents of their children in the ICU was 0.95. The reliability of the individual subscales I, II and II was equally high: 0.84; 0.95; and 0.93; respectively^11^.

*Coping Inventory for Stressful Situations (CISS)*

CISS created by Endler and Parker^4^, evaluates taking action in difficult and stressful situations. In our work, we used the Polish version of this scale in the adaptation of Strelau and Jaworowska^12^. CISS assesses stress coping strategies. It consists of 48 items evaluated on a five-point Likert scale: 1 – never, 2 – very rarely, 3 – sometimes, 4 – often, 5 – very often. It measures three styles of coping with stress: Task-Oriented Coping (TOC), Emotion-Oriented Coping (EOC) and Avoidance-Oriented Coping (AOC) in two subscales: engaging in substitute activities (subscale SA) and seeking social contacts (subscale SC). Each style includes 16 items, which means that the range of results for each style is from 16 to 80 points: for SA the range is 8–40 points, and for SC it is 5–25 points. The higher the score for a given style, the more often the person uses the strategies included in it. CISS has been normalized in a Polish adaptation (2003) using STEN norms for three age groups (16–24, 25–54, 55–79 years), with the scores: 1–4 (low) , 5–6 (average), and 7–10 (high)^11^. The Cronbach α coefficient for the items was in the range 0.72–0.92^11^.

### Variables

The parents in the study were actively involved in caring for their child (touch, feeding, routine care) who participated in kangaroo activities. Kangaroo care enables close contact of the bodies of a parent and a child, the ‘so-called’ skin-to-skin contact. In this article, we use both of these terms interchangeably, i.e. ‘kangaroo care’ (KMC) and skin-to-skin contact (SSC). Kangaroo Mother Care/skin-to-skin contact was always performed with the agreement of the parents. The parents who participated in our study started childcare with KMC, which has been used in the NICU in Poland. Kangaroo care/skin-to-skin is associated with direct contact of the bare child’s skin (undressed to a diaper) with the bare mother’s or father’s chest. During this type of care, a child could sleep or be breastfed. Time of KMC in the studied newborn group was varied from birth to the end of hospitalization in the ward, on average 1 month (up to the age of 1 week vs

over 1 week of the baby’s life: 65.1% vs 34.9%), in accordance with parent’s preferences, but not shorter than 1 hour.

### Statistical analysis

Statistical analysis was performed with Statistica 10.0 by StatSoft. The database was created in Microsoft Excel. The analysis of the variables was performed only with non-parametric tests. The ordinal nature of the numerical data detyermined the choice of this type of test. The differences in average numerical properties in the two populations were evaluated with the Mann-Whitney U test. The correlation of two numerical variables that did not meet the criteria of normality was calculated using Spearman’s rank correlation coefficient. The level of statistical significance adopted was at p<0.05.

### Ethical considerations

The study was approved by the Bioethics Commission at the Medical Faculty of the University of Rzeszow (protocol code No. 12/02/2013). This study was guided by ethical standards and national and international laws. All parents of the infants in NICU signed the consent form after receiving instructions regarding the possible risks and benefits and were granted privacy, confidentiality, and anonymity rights. The participants were free to stop participating at any stage of the experiment without giving reasons for their decision. Informed consent was obtained from all subjects involved in the study.

## RESULTS

### Characteristics of the study group

A total of 337 parents of premature infants were enrolled in the study. The majority were women (Female: 77.4%, n=261 vs Male: 22.6%, n=76). The mean age of the surveyed parents was 30.6 ± 5.1 years. In the surveyed group of parents, a majority declared higher education (Secondary: 43.6%, n=147 vs Higher: 56.4%, n=190) and all parents were professionally active i.e. they were employed under a contract of employment (n=337).

### The course of the present pregnancy

The course of current pregnancy in a small percentage was associated with the presence of diseases coexisting with pregnancy (28.5%, n=96 vs 71.5%, n=241) and the resulting complaints (38.6%, n=130 vs 61.4%, n=207).

Among diseases concomitant with pregnancy, the respondents indicated gestational diabetes (n=29), hypertension (n=27), thyroid disease (n=21), kidney disease (n=12) and gestational cholestasis (n=10). In other cases, the percentage of diseases associated with pregnancy did not exceed individual cases (1–4 cases) and concerned disorders of the hematopoietic system, deep vein thrombosis, cervical insufficiency and preeclampsia.

Among the predominant ailments in pregnancy, the respondents mentioned symptoms associated with developing pregnancy [i.e. nausea (n=32), bleeding from the genital tract (n=20), limb oedema (n=19), heartburn (n=16), malaise and headache (n=16), vomiting (n=13), back pain (n=9) and others (shortness of breath, dizziness, hemorrhoids] and symptoms accompanying diseases concomitant with pregnancy [urinary tract infection (n=7), pruritus (n=6), dizziness (n=6), dyspnea (n=4) and palpitations (n=2)].

### Clinical characteristics of the newborn

The majority of neonates were born between 29–36 week of pregnancy (gestational age <29 weeks: 26.9%, n=107; 29–32 weeks: 40.4%, n=161; 33–36 weeks: 32.7%, n=130), by cesarean section (82.5%, n=278 vs vaginal birth: 17.5%, n=59), in a moderate clinical condition at birth (Apgar mean score: 1st minute: 5.71 ± 3.16, n=304; 5th minute: 6.01 ± 3.31, n=217; 10th minute: 6.28 ± 3.49, n=203), moderately low birth weight (g) (<1499: 42.2%, n=168; 1500–2499: 40.4%, n=161; ≥2500: 17.4%, n=69). In this group of newborns, Kangaroo Mother Care /skin-to-skin was started from week 1 of life (1 week of baby’s life: 65.1%, n=194; over 1 week of baby’s life: 34.9%, n=104), more than an hour a day (daily: 56.0%, n=167; intermittently: 44.0%, n=131).

### Assessment of stress perception experienced by parents

Both in the overall assessment of stress and in individual subscales determining different types of stress, its greater intensity was observed in the group of examined mothers than in fathers (3.42 vs 2.99 points). These relationships were statistically significant at each subscale (Table 1).

**Table 1:** Summary of overall Parental Stressor Scale: Neonatal Intensive Care Unit (PSS:NICU) stress level scores among surveyed mothers and fathers, January–December 2016, Poland (N=337)

*PSS-NICU*	*Mothers (N=261)*	*Fathers (N=76)*	p*
	*Mean ± SD*	*Mean ± SD*	
Subscale I	2.90 ± 0.87	2.48 ± 0.80	<0.001
Subscale II	3.70 ± 0.93	3.41 ± 0.81	0.003
Subscale III	3.69 ± 0.96	3.08 ± 0.83	<0.001
The general level of stress in parents	3.42 ± 0.78	2.99 ± 0.71	<0.001

The degree of stress in subjects was determined on a 5-point Lickert scale (1 – not at all stressful; the experience did not cause you to feel upset, tense, or anxious, 2 – a little stressful, 3 – moderately stressful, 4 – very stressful, 5 – extremely stressful. Subscale I: sights and sounds related to therapeutic and diagnostic process. Subscale II: baby appearance and behavior. Subscale III: relationship with the baby and parental role.

* Mann-Whitney U test.

In the group of mothers practicing kangaroo care of children from 1 week of the child’s life, the results from subscale I (therapeutic and diagnostic process), subscale III (the role of parents during hospitalization and treatment of the child) and number on the entire PSS-NICU scale were significantly lower than in the group of mothers; kangaroo mother care over 1 week of the child’s life (subscale I: 2.80 vs 3.09; subscale III: 3.57 vs 3.91; total: 3.30 vs 3.32 points). Such relationships were not observed in the case of fathers practicing kangaroo care with their children (Table 2). The frequency of the kangaroo mother care implementation did not affect PSS-NICU scores in the group of mothers and fathers (Table 2).

**Table 2:** List of results of the Parental Stressor Scale: Neonatal Intensive Care Unit (PSS:NICU) of mothers and fathers giving KMC their children in NICU, January–December 2016, Poland (N=337)

*PSS-NICU and KMC*	*Mothers (N=261) Mean ± SD*		*p^a^*	*Fathers (N=76) Mean ± SD*		*p^a^*
	*Until the end of 1 week*	*Over the age of 1 week*		*Until the end of 1 week*	*Over the age of 1 week*	
Subscale I	2.80 ± 0.91	3.09 ± 0.69	0.024	2.60 ± 0.84	2.46 ± 0.73	0.518
Subscale II	3.60 ± 0.99	3.86 ± 0.75	0.107	3.56 ± 0.80	3.32 ± 0.77	0.283
Subscale III	3.57 ± 1.02	3.91 ± 0.73	0.024*	3.23 ± 0.86	2.96 ± 0.73	0.362
The general level of stress in parents	3.30 ± 0.82	3.62 ± 0.59	0.005*	3.13 ± 0.73	2.92 ± 0.65	0.411
***PSS-NICU and KMC frequency***	***Daily***	***Intermittently***		***Daily***	***Intermittently***	
Subscale I	2.90 ± 0.86	2.88 ± 0.85	0.777	2.46 ± 0.75	2.74 ± 0.85	0.163
Subscale II	3.71 ± 0.91	3.64 ± 0.94	0.596	3.55 ± 0.60	3.62 ± 0.80	0.607
Subscale III	3.64 ± 0.96	3.74 ± 0.93	0.446	3.20 ± 0.73	3.20 ± 0.93	0.876
The general level of stress in parents	3.40 ± 0.79	3.40 ± 0.75	0.851	3.07 ± 0.54	3.19 ± 0.78	0.596

The degree of stress in subjects was determined on a 5-point Lickert scale (1 – not at all stressful; the experience did not cause you to feel upset, tense, or anxious, 2 – a little stressful, 3 – moderately stressful, 4 – very stressful, 5 – extremely stressful. Subscale I: sights and sounds related to therapeutic and diagnostic process. Subscale II: baby appearance and behavior. Subscale III: relationship with the baby and parental role.

a Mann-Whitney U test.

* p<0.05.

### Stress management strategies

The most substantial strategy for coping with stress expressed in the surveyed group of parents giving KMC was Task-Oriented Coping (TOC), then Emotion-Oriented Coping (EOC) and Avoidance-Oriented Coping (AOC). The obtained results were transformed into values on a STEN scale (in accordance with Polish standards), allowing comparison of scales and subsets. Among the surveyed parents, TOC was on the high level (6.24 STEN), and EOC (5.02 STEN) on the average level. AOC (4.14 STEN) indicated a low level among all the surveyed parents. Similarly, seeking social contacts was at a low level, and involvement in substitute activities was on an average level (Table 3).

**Table 3:** STEN values for compilation of Coping Inventory for Stressful Situations (CISS) scores among all respondents, January–December 2016, Poland (N=337)

*CISS – STEN values*	*Mean*	*SD*
TOC	6.24	2.03
EOC	5.02	1.58
AOC	4.14	1.90
SC	4.53	2.10
SA	5.52	1.79

TOC: task-oriented coping. EOC: emotion-oriented coping. AOC: avoidance-oriented coping. SC: search for social contacts. SA: engaging in substitute activities. The degree of stress in subjects was determined on a 5-point Lickert scale (1 – not at all stressful; the experience did not cause you to feel upset, tense, or anxious, 2 – a little stressful, 3 – moderately stressful, 4 – very stressful, 5 – extremely stressful. The STEN scale is a standardized psychological test scale. The population mean is 5.5, and the standard deviation is 2. There are 10 units on the scale, range 1–10.

The results obtained on the CISS scale compiled among all the mothers and fathers studied, showed that the methods focused on emotions were more often the domain of women than men (43.32 vs 38.55 points, p<0.001); also, mothers were more likely than fathers to engage in surrogate activities (16.89 vs 15.53 points, p=0.016) (Table 4).

**Table 4:** Coping Inventory for Stressful Situations (CISS) scores among respondents taking into account gender, January–December 2016, Poland (N=337)

*CISS – Raw results*	*Mothers (N=261) Mean ± SD*	*Fathers (N=76) Mean ± SD*	*p^a^*
TOC	59.52 ± 8.89	61.17 ± 8.62	0.123
EOC	43.32 ± 9.72	38.55 ± 11.54	<0.001*
AOC	38.03 ± 8.02	37.46 ± 8.38	0.591
SC	16.07 ± 5.51	16.75 ± 5.87	0.384
SA	16.89 ± 3.40	15.53 ± 3.44	0.016*

TOC: task-oriented coping. EOC: emotion-oriented coping. AOC: avoidanceoriented coping. SC: search for social contacts. SA: engaging in substitute activities.

a Mann-Whitney U test.

* p<0.05.

In the group of mothers providing kangaroo care with children from 1 week of age, SA scores were higher than in the group of mothers who implemented kangaroo care with children over 1 week of age (16.72 vs 14.91 points, p=0.015). Such relationships were not found in the group of fathers (Table 5). The frequency of kangaroo mother care implementation did not affect the CISS scores in the group of mothers and fathers (Table 5).

**Table 5:** Coping Inventory for Stressful Situations (CISS) scale results among mothers and fathers with respect to the fact of undertaking KMC, January–December 2016, Poland (N=337)

*CISS and KMC initiation*	*Mothers (N=261) Mean ± SD*		*p^a^*	*Fathers (N=76) Mean ± SD*		*p^a^*
	*Until the end of 1 week*	*Over the age of 1 week*		*Until the end of 1 week*	*Over the age of 1 week*	
TOC	59.76 ± 8.68	59.16 ± 8.88	0.846	60.38 ± 9.53	61.32 ± 7.39	0.918
EOC	43.28 ± 9.89	42.97 ± 8.95	0.969	38.38 ± 12.71	37.95 ± 9.84	0.936
AOC	38.68 ± 8.12	36.66 ± 7.87	0.061	37.80 ± 9.57	37.26 ± 5.29	0.930
SC	17.01 ± 3.52	16.58 ± 3.34	0.222	15.64 ± 3.75	15.05 ± 2.72	0.355
SA	16.72 ± 5.58	14.91 ± 5.21	0.015*	16.82 ± 6.70	17.16 ± 4.44	0.490
***CISS and KMC frequency***	***Daily***	***Intermittently***		***Daily***	***Intermittently***	
TOC	59.82 ± 9.03	58.99 ± 8.31	0.610	61.44 ± 8.50	61.81 ± 9.09	0.956
EOC	43.23 ± 10.18	43.39 ± 8.52	0.723	35.48 ± 10.15	39.81 ± 11.26	0.149
AOC	37.85 ± 8.01	38.50 ± 8.19	0.536	35.89 ± 6.98	39.48 ± 9.23	0.137
SC	16.86 ± 3.47	16.88 ± 3.45	0.955	15.07 ± 3.74	16.26 ± 3.35	0.207
SA	16.08 ± 5.66	16.37 ± 5.29	0.610	15.48 ± 4.96	18.16 ± 6.67	0.178

TOC: task-oriented coping. EOC: emotion-oriented coping. AOC: avoidance-oriented coping. SC: search for social contacts. SA: engaging in substitute activities.

a Mann-Whitney U test.

* p<0.05.

## DISCUSSION

In our study, we noticed that mothers were more likely to experience higher stress levels, regardless of their subgroup level (I vs III). In turn, the tendency of fathers to experience a low level of stress, with a tendency to intensify it (3 points), occurred in the appearance of changes in the presentation and behavior of the child (subgroup II). The study’s novelty lies in assessing parents’ stress levels and how they cope with them during kangaroo care. To our knowledge, it was the first study of this type in Poland.

Our research confirms the results of other studies^13,14^, pointing to a moderately higher overall level of stress in the mothers’ group, and it was associated with the hospital environment, the sounds in the NICU, and the inability to perform the role of a parent. According to the results of our research and the cited studies of other authors, the higher level of stress caused by the unusual role of the parent and during the hospitalization and treatment of the child, seems to have its basis in the perception by mothers that their life is more stressful and challenging to predict, especially when it concerns a premature birth of a child and the resulting health problems^15^.

The results of Ionio et al.^16^ also indicate higher levels of maternal stress associated with spending more time in the NICU ward with the premature baby and feeling more guilty about it. These circumstances cause a greater emotional involvement of the mother in family life and her health, and the father’s emotions are centered around providing financial support to the immediate family^17,18^. Despite the lower stress levels of the fathers, due to the baby’s appearance and the surrounding equipment, their emotional commitment to baby care and fulfilling the role of a parent are maintained^19^.

Slightly different observations from our stress-related parents were made by the Ionio et al.^16^. Although mothers reported higher stress levels than fathers, the difference was evident in the subscale associated with physical stimuli related to the child’s appearance and behavior^20^.

We also noticed that the time of commencement of KMC by parents significantly reduces the stress level during the child’s hospitalization. Our results seem to confirm the observations of Miles et al.^21^ and Young Seidman et al.^22^. In addition, the child’s hospitalization in the NICU and the resulting stress may limit the parent’s ability to connect with the child^23^.

In the face of so many stressors for a parent, we decided to check how the KMC method, widely recognized in Poland and worldwide, would affect the level of stress and ways of coping with it by mothers and fathers. Especially that parents who want to touch their child (KMC) often experience in this situation internal conflicts such as ‘I want, but I am afraid’, resentment/disgust, and helplessness, even though they feel that they are responsible for their child for the first time^24^.

This situation may be related to the strengthening of parental feelings and adaptation to the ‘new’ role of the KMC parent^25^. In addition, the clinical benefits in child development observed during this time will be conducive to a sense of parental control in child hospitalization, building their confidence and acquiring new competencies^26,27^.

In our research, we also noticed that parents giving KMC to their children experience different levels of stress intensity depending on the time of initiation of this type of contact. Although the level of stress differed in the group of mothers and fathers, the only significantly lower differences were observed in the subscale of the therapeutic and diagnostic process and the role of the parent during the hospitalization of the child in the group of women giving KMC at 1 week of the child’s life compared to mothers initiating such initiatives over 1 week and hospitalization of the premature baby^28^. Although our research did not observe this type of relationship in the group of fathers, the research of Lamb and Lewis^29^ draws attention to the fact that fathers are more susceptible to physical contact with their child during KMC interaction than mothers^30^.

Our research goal was also to identify strategies for coping with the stress in the mothers and fathers when experiencing it. Coping has been defined as the constantly changing cognitive and behavioral efforts to manage specific external and/or internal demands that have been evaluated as taking up or exceeding the resources of the person. Research recognizes two major functions of coping: regulating stressful emotions and altering the person–environment relation causing the stress^31^.

Problem-centered coping involves cognitive and behavioral attempts to modify or eliminate a stressful situation, with the main focus being on tasking or planning to resolve the situation. In contrast, emotion-centered coping involves trying to regulate the emotional responses triggered by a situation^31^, which is less effective, and avoidance-oriented coping focuses on ways to avoid tackling current problems^11^. In order to investigate how to deal with the stress of parents of premature babies, we used CISS^4^, which is recommended in Poland.

We observed that accompanying the infant and engaging in the caring, activates defence mechanisms in parents including emotional assessment and management, which are important in stress reduction. This situation is an important element in reducing stress; however, specific stress strategies are influenced by individual personality traits, sociodemographic variables (age, sex, education level), current psychophysical condition^25^ and history of birth complications^24^.

In the surveyed group of mothers and fathers, the disposition to struggle with stress was TOC. In turn, the NICU environment and infant’s reactions observed during skin-to-skin contact resulted in higher stress severity experienced by parents. They were similar in the case of parents focused on EOC and AOC during KMC.

Based on the correlation analysis obtained, the psychological characteristics and behavior of fathers under challenging situations allow them to react more flexibly in dealing with a child’s prematurity^31^. The results of our research indicate some similarities in the way the parents giving KMC deal with stress. They suggest that focusing on one of the avoidance strategies, including emotions, can facilitate better adaptation to stressful situations than in TOC^32^.

Our research confirmed that mothers, more often than fathers, felt stress when seeing and hearing medical equipment used in the child’s therapeutic process, observing their child’s appearance and behavior, as well as during parental interactions with the child.

The results of our research compared to Polish and foreign studies are varied^31-33^, and they indicate that women more often focus on emotions and avoidance than men, who prefer TOC, but in both cases, the severity of the disorders in the baby’s development determines them^34^. In this situation, fathers often need time to adapt emotionally to the role of father^24^, which is greater the more stressed they are^35^. Parents’ reactions to difficult situations due to the hospitalization of a baby in NICU will undoubtedly depend on sex, social role, individual psychological resources and coping strategies^36^.

### Limitations

Our study has some limitations. A limitation of the study is the small sample of the parents studied and only a onetime assessment of the stress level of mothers and fathers. In addition, it would be worth continuing long-term research in a group of parents in different cultural circles comparing the stress level and coping strategies at different stages of the child’s development. We are aware that our research results represent only a small part of the impact of skin-to-skin contact on the level of stress experienced by parents and ways to counteract it, and they require future multi-center randomized trials to confirm the results.

## CONCLUSIONS

The stress from each of the sources under consideration was stronger among mothers. Also, in the group of women practicing initiation of kangaroo mother care at 1 week of their children’s life, the overall stress score, subscale I and subscale III, was significantly lower. In contrast, the kangaroo mother care intensity did not affect the PSS:NICU scores in the group of mothers and fathers studied. Stress experienced by mothers and fathers results in many coping strategies. The dominant mode of coping with stress in mothers and fathers was task-oriented coping but focusing on one of the Avoidance-Oriented coping strategies –the search for social contacts in mothers and Avoidance-Oriented Coping in fathers.

## Data Availability

The data supporting this research is available from the authors on reasonable request.
